# Bidirectional evaluation of canal transportation, centering ability and curvature changes of three NiTi rotary systems using cone beam computed tomography (invitro study)

**DOI:** 10.1186/s12903-025-07414-z

**Published:** 2025-12-17

**Authors:** Ahmed A. Soliman, Raef A. Sherif, Amr M. Abdallah, Ahmed M. Mobarak

**Affiliations:** 1 Post Graduate Student Conservative Dentistry Department, Faculty of Dentistry, Alexandria University, Alexandria, Egypt; 2 Professor of Endodontics Conservative Dentistry Department, Faculty of Dentistry , Alexandria University, Alexandria, Egypt; 3 Dean of Faculty of Dentistry, Pharos University, Alexandria, Egypt; 4 Associate Professor Conservative Dentistry Department, Faculty of Dentistry, Alexandria University, Alexandria, Egypt

**Keywords:** Angle of curvature, Canal transportation, CBCT, Centering ability, Curved canal, HyFlex EDM one, RCS rainbow one, Shaping ability, Zarc blueshaper

## Abstract

**Background:**

The main aim of root canal treatment is to clean and shape root canals while preserving the original canal anatomy. Even with improvements in the manufacturing of NiTi files, maintaining the original canal anatomy in curved root canals is a challenge.

**Aim of the study:**

This study aimed to compare the shaping ability of three different NiTi files via CBCT to assess mesiodistal and buccolingual canal transportation, canal centering ability, and canal straightening in severely curved canals.

**Materials and methods:**

Thirty-six human extracted mandibular molars were used in this study, and the samples were randomly divided into three equal groups (*n* = 12) according to the files used for root canal instrumentation. Preoperative CBCT scans were performed to evaluate the canal position at three different levels: apical, middle, and coronal. Group I: mesiobuccal (MB) canals of each molar were instrumented with Zarc BlueShaper files (Zarc4Endo, Gijon, Spain) up to Z4 file (25/~), Group II: MB canals were instrumented with RCS Rainbow One file (RAMO MEDICAL, Suzhou, China) of size (25/0.06), and Group III: MB canals were instrumented with HyFlex EDM One file (25/~) (Coltene/Whaledent AG, Alstätten, Switzerland). Measurements were performed on Postoperative CBCT scans of instrumented canals to evaluate the canal transportation, centering ability and straightening at the apical, middle and coronal levels.

**Results:**

HyFlex EDM One demonstrated the most favorable shaping performance, showing minimal transportation, high centering, and least curvature alteration. These outcomes were followed by Zarc BlueShaper and subsequently by RCS Rainbow One. A statistically significant difference was observed in mesiodistal overall centering ability, where RCS Rainbow One exhibited less centering ability than HyFlex EDM One (*P* = 0.048). However, no statistically significant differences were detected among the three groups regarding mesiodistal canal transportation (*P* = 0.14), buccolingual canal transportation (*P* = 0.39), buccolingual centering ability (*P* = 0.6), or curvature change (*P* = 0.42).

**Conclusions:**

Within the limitations of this in vitro study, all the tested rotary systems demonstrated clinically acceptable performance in shaping curved root canals, despite minor differences in canal transportation, centering ability, and canal straightening.

**Supplementary Information:**

The online version contains supplementary material available at 10.1186/s12903-025-07414-z.

## Background

Chemomechanical preparation is considered the most crucial stage of endodontic treatment as it not only removes the bacterial infection of the root canal, which is the cause of apical periodontitis, but also shapes the tooth to accommodate a suitable filling [1, 2].

Shaping curved root canals and teeth with complex internal anatomy remains a challenge for clinicians [3, 4]. The cutting action of instruments exerts a greater effect on some canal walls than on others. Certain regions may remain unprepared, increasing the risk of procedural errors such as transportation, ledge formation, or perforation. Cleaning and disinfection are also negatively impacted. This may result in bacterial persistence in the unprepared area of the canal system, which could jeopardize the treatment success for teeth with apical periodontitis. Maintaining the apical foramen position and the original canal configuration is essential during shaping [5–7].

Since the introduction of nickel-titanium (NiTi) rotary systems, endodontic instrumentation has become faster and safer than with stainless steel files owing to the super elasticity of NiTi instruments, which favors preservation of the original canal anatomy [8].

Even though these instruments show lower rates of procedural errors such as zips and perforations, certain dentinal areas remain unprepared due to the unfavorable shape memory effect of NiTi files. This may cause asymmetric dentin removal weakening the root and increasing the risk of strip perforation, potentially leading to treatment failure [9, 10].

Three-dimensional root canal preparation is still a significant challenge for endodontists, despite advancements in the manufacturing process of NiTi rotary instruments. To address this, instruments with diverse geometries and surfaces have been introduced, making canal shaping more predictable [8]. A variety of rotary systems featuring distinctive designs in tip, taper, pitch, rake, and helical angles, along with thermomechanical treatments to improve mechanical properties, increase fatigue resistance, and enhance deformability through controlled-memory (CM) technology for better canal curvature adaptation, have recently been brought to market [11–13].

The newly introduced BlueShaper file (Zarc4Endo, Gijon, Spain) is the first system to incorporate 2 alloys: the pink alloy to increase torsional resistance in Z1 file and the blue alloy that enhances flexibility and cyclic fatigue resistance while respecting the original canal anatomy. The system features a convex triangular cross section, and consists of 3 shaping files (Zx-Z1-Z2) and 5 finishing files (Z3-Z4-Z5-Z6-Z7) providing a final preparation with a regressive taper and tip sizes of 20,25,30,35 or 40 [14, 15].

The RCS Rainbow One file (RAMO MEDICAL, Suzhou, China) has a slim S-shaped cross section and special heat-treated technique. According to the manufacturer, the variation in color is attributed to the presence of a diamond-like carbon (DLC) coating During manufacturing, the instruments are placed in a vacuum furnace, where they undergo solid-state ionization through a graphite target, resulting in the deposition of a thin DLC film on the surface. This coating enhances surface smoothness, hardness, and chemical resistance, thereby improving fracture resistance, torsional strength, and flexural properties, as well as increasing cutting efficiency by more than 150%. The file has a variable pitch design and operates as a single file system with different file sizes and tapers according to the canal anatomy [16, 17].

HyFlex EDM One file (Coltene/Whaledent AG, Alstätten, Switzerland) is a controlled-memory (CM) NiTi system produced through a unique thermomechanical process that eliminates traditional NiTi shape memory, making the files extremely flexible [18]. Its superior properties are attributed to a breakthrough technology called “Electrical Discharge Machining”. This innovative manufacturing process uses spark erosion to harden the surface of the NiTi file, resulting in superior fracture resistance and improved cutting efficiency. The file features a variable cross section design; an almost triangular cross section at the top, a trapezoidal cross section at the middle and a quadratic cross section at the tip, and CM allows superior canal tracking [18, 19].

The assessment of an instrument’s ability to remain in the center of the canal during instrumentation is performed in several ways; there is no standard method for doing so. These methods include computed tomography (CT), cone beam CT (CBCT), micro-CT, stereomicroscopy, radiography, scanning electron microscopy, and histologic Sects [20, 21]. CBCT is a non-invasive 3D imaging modality that allows assessment of canal shape and position before and after instrumentation at different levels [22–24].

The null hypothesis stated that there is no significant difference in the shaping ability of the three NiTi rotary file systems in curved mesiobuccal (MB) canals of mandibular molars. The aim of this study was to compare the shaping ability of Zarc BlueShaper, RCS Rainbow One file, and HyFlex EDM One file in severely curved MB canals of mandibular molars via CBCT.

- Primary objective: To compare mesiodistal and buccolingual canal transportation and centering ability after root canal preparation via CBCT among the three groups.
- Secondary objective: To evaluate canal straightening by measuring the angle of curvature before and after root canal preparation.

## Materials and methods

### Study setting

- The samples were prepared and examined at the Conservative Department Laboratory at the Faculty of Dentistry, Alexandria University.
- The CBCT images were obtained at I-Scan dental radiography center.

This study was approved by the Institutional Review Board of the Faculty of Dentistry, Alexandria University (IORG:0008839, approval no. 0622-02/2023). Sample size was estimated assuming an alpha error of 5% and a study power of 80%. The mean (SD) canal transportation was 0.40 (0.22) for HyFlex file, 0.52 (0.22) for ProTaper file, like Zarc BlueShaper [25], and 0.11 (0.003) for the One Curve file, similar to RCS Rainbow One file [26]. Based on the difference between independent means using F test and the highest SD = 0.22 [25] to ensure enough study power. The sample size was calculated to be 11 samples per group, increased to 12 samples to make up for processing errors. The total sample size was 36 teeth (12 per group × 3 groups). Sample size estimation was based on Rosner’s method [27] and calculated using G*Power version 3.1.9.7 [28].

## Materials

Thirty-six human permanent mandibular molars were collected from the outpatient clinic of the Oral and Maxillofacial Surgery Department, Faculty of Dentistry, Alexandria University, which were extracted for orthodontic or periodontal reasons. Radiographs were taken for all the teeth to check for inclusion criteria.

The teeth selected were fully developed mandibular molars with 2 separate mesial and distal roots with completely formed apices, two separate mesial canals with 2 separate foramina (class IV Vertucci) [29], canal curvature between 20° and 50° (Schneider’s method) [30], and the initial file in the MB canal shouldn’t exceed #15 K file.

The teeth cleaned with 0.5% sodium hypochlorite to remove debris, tissue fragments, and calculus from the root surface, then rinsed and stored in distilled water until use.

All teeth were radiographed via Vatech EzSensor Classic imaging RVG (Hwaseong-si, Gyeonggi-do, Korea) preoperatively to detect any calcifications or intrapulpal and intracanal abnormalities and to assess the degree of curvature of the mesial roots.

The teeth were randomly numbered by random sequence generation of numbers 1–36, and the samples were randomly divided into three equal groups according to the rotary files system used in the root canal preparation (*n* = 12).

- Group I: Zarc BlueShaper.
- Group II: RCS Rainbow One file.
- Group III: HyFlex EDM One file.

### Mounting the samples

Nine prefabricated rectangular acrylic molds with dimensions of 9 × 2 cm were used. Putty condensation silicone impression material was inserted inside the mold and four teeth were positioned in each mold before setting of condensation silicone to standardize their position during the preoperative and postoperative CBCT scans [31].

Each mold was labeled according to the sample group and the rotary file system used.

- Group I: Molds (A, B& C).
- Group II: Molds (D, E& F).
- Group III: Molds (G, H& I).

### Preinstrumentation CBCT evaluation

Primary CBCT images were obtained before instrumentation via the cone beam 3D imaging system. The molds containing the samples were mounted on a CBCT scanner for pre-instrumentation imaging. CBCT images were acquired with a J-Morita R100 device (J-Morita, Kyoto, Japan). The scan was performed with a field of view of width 100 mm X height 40 mm. The voxel size was set to 0.125 mm The tube voltage was 75 kV and 1 mA and an exposure time of 9.4 s. Axial slices were obtained at three, six, and nine millimeters from the apex of the MB root canal via OnDemand 3D software (Cyber Med, USA) to represent the apical, middle, and coronal thirds respectively. At each level, the distances between the mesial wall of the MB root canal wall and the outer root surface mesially, the distal wall of the MB root canal and the distal root surface, the buccal wall of the MB root canal and the buccal root surface, and the lingual wall of the MB root canal and the lingual root surface were measured.

### Sample preparation

All teeth were decoronated to a standard length of 19 mm via a double-faced diamond disc on a low-speed handpiece to standardize the working length and eliminate any confounders that might affect the shaping procedure. The access cavity was made using #4 diamond round bur (Dentsply Sirona maillefer, Ballaigus, Switzerland) on a high-speed handpiece with water coolant until penetration into the pulp chamber, then access refinement was performed via an endo-Z bur for finishing and flaring the access cavity. The MB canal orifice was located using an endodontic explorer. K-file #10 (Dentsply Sirona maillefer, Ballaigus, Switzerland) was used to check the patency of the MB canal and working length determination by inserting #10 K-file until it was visible from the root apex and measuring its length reduced by 1 mm. A glide path was made using #10 and #15 K-files using chelating EDTA gel and irrigation with 2.5% sodium hypochlorite with a side-vented 30-gauge endodontic irrigation tip to the working length. The root canal instrumentation of the MB root canal was then performed according to the group.

### Instrumentation procedures

#### Group I: Zarc Blueshaper

Using the Endo Gold endo motor (Woodpecker, China), instrumentation of the MB canal to the full working length was performed with Zarc BlueShaper files, with the motor adjusted at a speed of 500 rpm with continuous rotation and a torque of 4 N as recommended by the manufacturer [15]. Using EDTA gel and sodium hypochlorite irrigation (2.5%), the canal was instrumented to the full working length with the sequence Z1(14/~), Z2(17/~), Z3(19/~), and then Z4(25/~). The canal was irrigated, recapitulated, and patency was checked between each file.

#### Group II: RCS Rainbow one file

The endo motor was adjusted at a speed of 350 rpm and a torque of 1.5 N, and the RCS Rainbow open file (17/08) was used in picking motion without pressure for coronal flaring of the canal [17]. Then, the RCS Rainbow One file (25/0.6) was used at a speed of 400 rpm and a torque of 2 N, coated with EDTA gel lubricant, with intermittent irrigation until the working length was reached to finish the MB canal shaping.

#### Group III: HyFlex EDM one file

The endo motor was adjusted at a speed of 400 rpm and a torque of 2.5 N in continuous rotation according to the manufacturer’s instructions [3]. HyFlex EDM One file (25/~) was coated with EDTA gel lubricant and introduced into the MB canal without pressure till reaching the middle third of the canal. When resistance was encountered, the file was withdrawn, the flutes were cleaned, the canal was irrigated, and recapitulation and patency were checked via #10 and #15 St-St files. Then, the HyFlex EDM One file was reinserted into the canal until the desired working length was reached.

### Postoperative CBCT evaluation

After complete instrumentation, all teeth were cleaned and irrigated with (2.5%) sodium hypochlorite and set back to their place in the mold. The molds were mounted for postoperative CBCT scanning with the same settings as those used for the preoperative scan.

### Canal transportation and centering ability evaluation

**Fig. 1:**
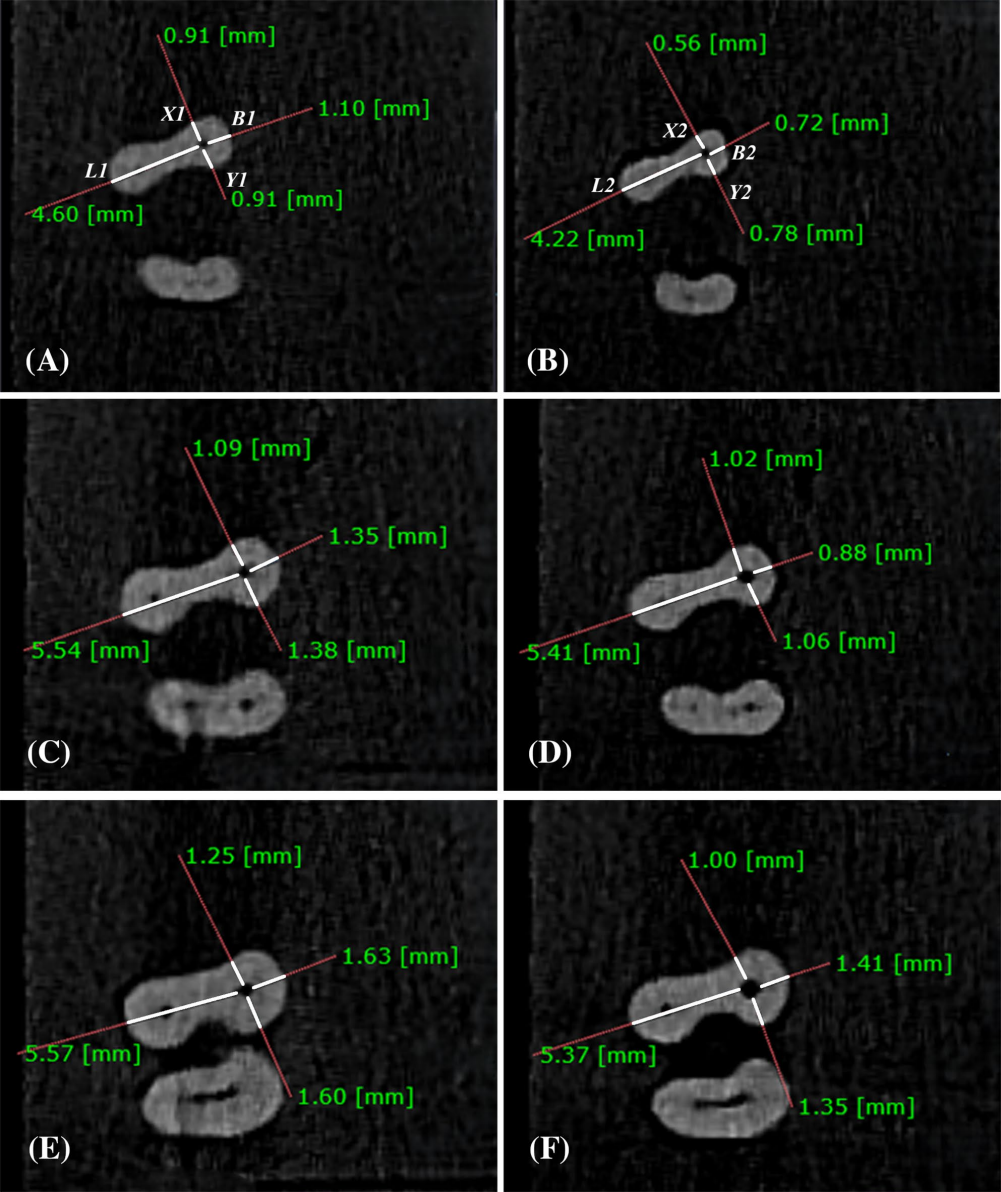
Showing CBCT images analysis of one of the samples at three, six- and nine-mm levels. **A **& **B** Axial sections of pre and post instrumentation measurement at three mm level, respectively. **C **& **D** Axial sections of pre and post instrumentation measurement at six mm, respectively. **E **& **F** Axial sections of pre and post instrumentation measurement at nine mm, respectively

The evaluation of canal transportation and centering ability was conducted via the Gambill method [21–23]. Canal transportation was assessed at three distinct levels from the tooth apex: apical (3 mm), middle (6 mm), and coronal (9 mm), as shown in (Fig. 1). The mesiodistal canal transportation of the MB canal was determined via the following equation:

$$(X1-X2)\;-\;(Y1-Y2)$$

where* X1* represents the distance from the mesial root surface to the mesial wall of the uninstrumented MB canal, *X2* denotes the same distance after instrumentation, *Y1* represents the distance from the distal root surface to the distal wall of the uninstrumented MB canal, and *Y2* denotes this distance after instrumentation [20, 22, 24].

Similarly, buccolingual canal transportation was calculated using the following formula:

$$(B1-B2)\;-\;(L1-L2)$$

where *B1* represents the distance from the buccal root surface to the buccal wall of the uninstrumented MB canal, *B2* is the same distance after instrumentation, *L1* represents the distance from the lingual root surface to the lingual wall of the uninstrumented canal, and *L2* denotes this distance after instrumentation [32].

If the result is “0”, this indicates that no canal transportation occurred, negative results indicate canal transportation toward the distal and the lingual sides, whereas positive results indicate deviation toward the mesial and buccal sides.

The canal centering ability was calculated via the following equations: [33]

$$(X1-X2)/(Y1-Y2)\;or\;(Y1-Y2)/(X1-X2)$$

$$(B1-B2)/(L1-L2)\;or\;(L1-L2)/(B1-B2)$$

Given that the fraction with the smaller value was used for statistical analysis, a result of “1” indicates perfect centering of the canal preparation [21] and a result of “0” indicates complete decentralization.

### Canal straightening evaluation

**Fig. 2:**
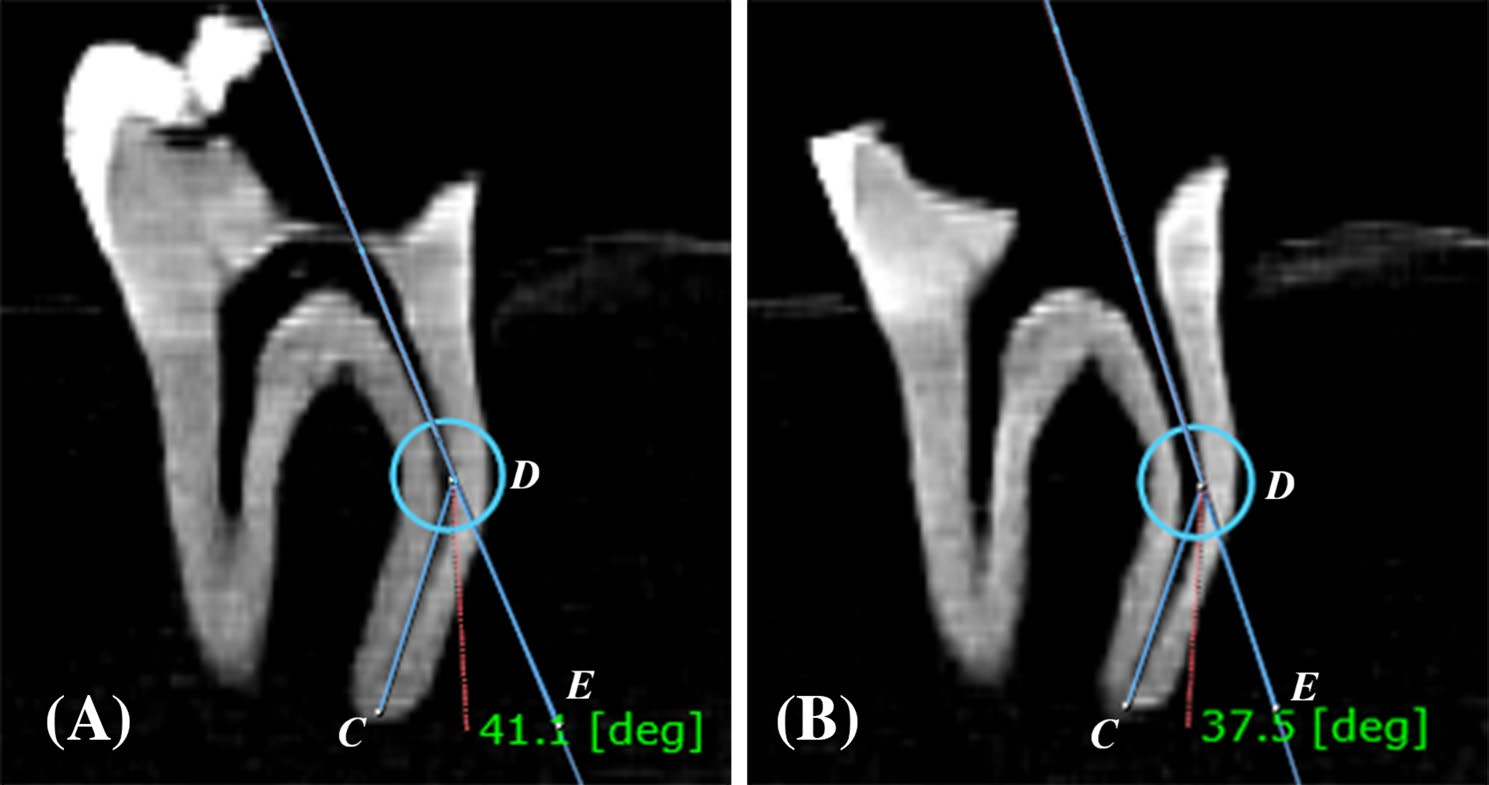
Representing CBCT sagittal view to measure Schneider angle of curvature CDE. **A** preoperative angle of curvature. **B** postoperative angle of curvature

The evaluation of canal straightening was conducted by comparing the angle of curvature of the root canals before and after instrumentation, following Schneider’s method [30, 34]. Two straight lines were used for this analysis: the first line extends from the canal orifice to the point where the canal direction changes, whereas the second line starts at the apical foramen and connects to the same deviation point. The angle formed by the intersection of these two lines was measured and referred to as the angle of curvature, as shown in (Fig. 2). The preoperative and postoperative angles were then compared to assess the degree of canal straightening [32, 35, 36].

### Statistical analysis

The normality of the continuous data was tested using the Kolmogrov-Smirnov and Shapiro-Wilk tests. Continuous data are presented as the mean and standard deviation (SD). Differences between Zarc BlueShaper, RCS Rainbow One, and Hyflex EDM One files in canal transportation, centring ability, and canal straightening were tested via the Kruskal-Wallis test followed by the Bonferroni post-hoc adjustment when significant difference was found. The significance level was set at (*p* < 0.05). The data were analysed via SPSS version 23.0 for Windows (SPSS Inc., Chicago, USA).

## Results

### Comparison of mesiodistal canal transportation among the three study groups at 3, 6, and 9 mm and overall levels** (**Table 1**)**

At *3 mm*, HyFlex EDM One group had more canal transportation than the other groups, with the highest mean value (0.04), followed by both Zarc BlueShaper (−0.02) and RCS Rainbow One files (0.02). However, there was no statistically significant difference among the groups (*P* = 0.44).

At the *6 mm* level, the RCS Rainbow One system exhibited the lowest mean (−0.01), followed by the Zarc BlueShaper (0.03) and HyFlex EDM One groups (0.06). Also, the differences were not statistically significant (*P* = 0.57).

At *9 mm*, HyFlex EDM One had the least canal transportation (−0.01) followed by Zarc BlueShaper (−0.11) and RCS Rainbow One groups (−0.17), but this difference was not statistically significant (*P* = 0.13).

*Overall*, the mean mesiodistal canal transportation values were (0.03) for HyFlex EDM One, (−0.03) for Zarc BlueShaper, and (−0.05) for RCS Rainbow One, with no statistically significant difference among the three groups (*P* = 0.14).

**Table 1:** Differences in mesiodistal canal transportation between the file types across the root sites (*n* = 36)

Root site		Zarc BlueShaper *n* = 12	RCS Rainbow One *n* = 12	HyFlex EDM One *n* = 12	*p* value
3 mm	Mean (SD)	−0.02 (0.12)	0.02 (0.12)	0.04 (0.10)	0.44
6 mm	Mean (SD)	0.03 (0.19)	−0.01 (0.18)	0.06 (0.14)	0.57
9 mm	Mean (SD)	−0.11 (0.28)	−0.17 (0.17)	−0.01 (0.10)	0.13
Total root	Mean (SD)	−0.03 (0.21)	−0.05 (0.18)	0.03 (0.12)	0.14

### Comparison of buccolingual canal transportation among the three study groups at 3, 6, and 9 mm and overall levels (Table 2)

At the *3 mm* level, HyFlex EDM One presented the lowest buccolingual transportation (0), followed by Zarc BlueShaper (−0.02) and then RCS Rainbow One (−0.04). No statistically significant differences were detected among the groups (*P* = 0.89).

At *6 mm*, HyFlex EDM One continued to exhibit the lowest transportation (0.02), followed by Zarc BlueShaper (−0.03), whereas the RCS Rainbow One group had the highest transportation value (−0.07). These differences were not statistically significant (*P* = 0.80).

At *9 mm*, the Zarc BlueShaper group recorded the lowest buccolingual transportation (−0.06), followed by HyFlex EDM One (−0.11), and RCS Rainbow One showing the highest value (−0.23). The differences were not statistically significant (*P* = 0.15).

*Overall*, HyFlex EDM One achieved the lowest mean of buccolingual transportation (− 0.03), indicating superior canal anatomy preservation. This was followed by Zarc BlueShaper (− 0.04) and RCS Rainbow One (− 0.11). The differences among the groups were not statistically significant (*P* = 0.39).

**Table 2:** Differences in buccolingual canal transportation between the file types across the root sites (*n* = 36)

Root site		Zarc BlueShaper *n* = 12	RCS Rainbow One *n* = 12	HyFlex EDM One *n* = 12	*p* value
3 mm	Mean (SD)	−0.02 (0.11)	−0.04 (0.21)	0.00 (0.14)	0.89
6 mm	Mean (SD)	−0.03 (0.24)	−0.07 (0.37)	0.02 (0.20)	0.80
9 mm	Mean (SD)	−0.06 (0.31)	−0.23 (0.21)	−0.11 (0.18)	0.15
Total root	Mean (SD)	−0.04 (0.23)	−0.11 (0.28)	−0.03 (0.18)	0.39

### Comparison of mesiodistal centering ability at 3, 6, and 9 mm and overall levels among the three studied groups (Table 3)

At the *3 mm* level, Zarc BlueShaper exhibited the highest centering ability (0.59), followed by HyFlex EDM One (0.52) and RCS Rainbow One (0.35). The differences were not statistically significant (*P* = 0.21).

At *6 mm*, both HyFlex EDM One and Zarc BlueShaper recorded similar mean values (0.58), while RCS Rainbow One demonstrated lower centering ability (0.48). No statistically significant differences were observed (*P* = 0.58).

At *9 mm*, HyFlex EDM One had the highest centering ability (0.63), followed by RCS Rainbow One (0.39) and then Zarc BlueShaper (0.38). The differences among the groups were not statistically significant (*P* = 0.07).

*Overall*, HyFlex EDM One exhibited the highest mean mesiodistal centering ability (0.57), followed by Zarc BlueShaper (0.52), while RCS Rainbow One showed the lowest centering value (0.41). A statistically significant difference was found between HyFlex EDM One and RCS Rainbow One (*P* = 0.048).

**Table 3:** Differences in the mesiodistal canal centering ability between the file types across the root sites (*n* = 36)

Root site		Zarc BlueShaper *n* = 12	RCS Rainbow One *n* = 12	HyFlex EDM One *n* = 12	*p* value
3 mm	Mean (SD)	0.59 (0.37)	0.35 (0.27)	0.52 (0.32)	0.21
6 mm	Mean (SD)	0.58 (0.27)	0.48 (0.26)	0.58 (0.29)	0.58
9 mm	Mean (SD)	0.38 (0.30)	0.39 (0.35)	0.63 (0.26)	0.07
Total root	Mean (SD)	0.52 (0.32)^ab^	0.41 (0.29)^a^	0.57 (0.29)^b^	0.048*

^a-b^Different letters denote statistically significant difference between files in the total root lengthusing Bonferroni adjustment

^*^Statistically significant at *p* < 0.05

### Comparison of buccolingual centering ability at 3, 6, and 9 mm and overall levels among the three studied groups (Table 4)

At *3 mm*, Zarc BlueShaper achieved the highest centering ability (0.7), followed by RCS Rainbow One (0.57) and HyFlex EDM One (0.43). The differences were not statistically significant (*P* = 0.09).

At the *6 mm* level, RCS Rainbow One presented the highest centering ability (0.53), followed by HyFlex EDM One (0.51) and then Zarc BlueShaper (0.43). No statistically significant differences were observed (*P* = 0.67).

At *9 mm*, Zarc BlueShaper again recorded the highest centering ability (0.49), followed by HyFlex EDM One (0.48) and RCS Rainbow One (0.34). These differences were not statistically significant (*P* = 0.29).

*Overall*, Zarc BlueShaper had the highest mean buccolingual centering ability (0.54), followed by RCS Rainbow One (0.48) and HyFlex EDM One (0.47). The differences were not statistically significant (*P* = 0.60).

**Table 4:** Differences in buccolingual canal centering ability between the file types across the root sites (*n* = 36)

Root site		Zarc BlueShaper *n* = 12	RCS Rainbow One *n* = 12	HyFlex EDM One *n* = 12	*p* value
3 mm	Mean (SD)	0.70 (0.19)	0.57 (0.34)	0.43 (0.29)	0.09
6 mm	Mean (SD)	0.43 (0.15)	0.53 (0.34)	0.51 (0.34)	0.67
9 mm	Mean (SD)	0.49 (0.31)	0.34 (0.34)	0.48 (0.25)	0.29
Total root	Mean (SD)	0.54 (0.25)	0.48 (0.35)	0.47 (0.29)	0.60

### Comparison of canal straightening and percent angle change among the three studied groups (Table 5)

Assessment of canal straightening, as determined by the difference in pre- and postoperative angles of curvature using Schneider’s technique, revealed a reduction in angle values in all groups. However, no statistically significant differences were observed among the three groups (*P* > 0.05).

Zarc BlueShaper exhibited the highest mean change in angle (4.34°) and percent change (10.69%), indicating greater canal straightening. RCS Rainbow One followed with a mean angle change of (3.42°) and a percentage change of (10.13%). HyFlex EDM One showed the least amount of change, with a mean angle reduction of (2.62°) and a percentage change of (7.74%), suggesting better preservation of the original canal curvature.

**Table 5:** Differences in Canal Straightening between the file types (*n* = 36)

Variables		Zarc BlueShaper *n* = 12	RCS Rainbow One *n* = 12	HyFlex EDM One *n* = 12	*p* value
Canal straightening (Preoperative- Postoperative angles)	Mean (SD)	4.34 (2.87)	3.42 (2.08)	2.62 (1.13)	0.28
Percent of change	Mean (SD)	10.69 (6.68)	10.13 (6.18)	7.74 (2.88)	0.42

## Discussion

The primary objective of root canal shaping extends beyond mechanical enlargement of the canal. It encompasses the thorough removal of organic and inorganic debris while preserving the canal’s natural curvature and continuous taper from the apex to the coronal region which is fundamental to long-term endodontic success. Preservation of the dentinal wall thickness is critical, especially in curved root canals, to maintain the structural integrity of the tooth and reduce the risk of vertical fracture [37]. Therefore, shaping systems should be evaluated not only for cutting efficiency but also for their capacity to minimize canal aberrations, particularly transportation and straightening [38].

Shaping irregularities, such as canal transportation and inadequate centering leading to ledge formation, zipping, or even perforation, especially when files are not adequately flexible to conform to complex canal anatomy, can result in adverse clinical consequences. Canal transportation can lead to areas of uninstrumented canal walls, increasing the risk of persistent infection. In addition, such deviations may impair obturation quality, reduce the sealing efficiency of root canal filling, and increase susceptibility to fracture [8].

Technological advancements in nickel-titanium (NiTi) rotary instruments have aimed to address these issues through modifications in file design, alloy composition, and kinematic behavior [8, 12]. File geometry, including cross-sectional shape, taper, tip design, and helical angle, plays a critical role in determining file behavior within the canal. Off-centered cross-sections, progressive tapers, and reduced core masses help minimize the screw-in effect, distribute stress more uniformly, and improve shaping outcomes, especially in curved canals [39, 40].

In this study, the shaping ability and canal straightening of three thermally treated rotary NiTi systems: Zarc BlueShaper, RCS Rainbow One, and HyFlex EDM One, were evaluated via cone beam computed tomography (CBCT). Measurements were recorded at 3 mm, 6 mm, and 9 mm from the apex in both mesiodistal and buccolingual planes. These systems differ in design, metallurgy, and manufacturing technique, offering an ideal comparison for analyzing shaping performance in anatomically complex root canals.

The Zarc BlueShaper is engineered with a triple helix cross-section, a regressive taper, and a blue heat-treated NiTi alloy. The file’s off-centered design and alternating cutting edges reduce torsional stress and promote controlled dentin removal. This results in improved flexibility and a reduced screw-in effect, facilitating more conservative and centered canal preparation. The heat treatment enhances the fatigue resistance of the file and allows a mixed austenite-martensite phase transformation at body temperature, contributing to its shape memory and adaptive behavior [15].

The RCS Rainbow One is a single-file system manufactured from heat-treated NiTi alloy. It features an S-shaped cross-section and a DLC coating, which is reported to improve surface hardness and cutting efficiency [41]. The file’s variable pitch and positive rake angle reduce apical pressure and debris compaction while enhancing coronal evacuation. These features, combined with continuous rotation kinematics, are intended to optimize cutting performance while preserving canal centrality [17].

HyFlex EDM One integrates a changing cross-sectional geometry along its length, transitioning from square to trapezoidal to triangular forms. This design facilitates centered progression through curved canals while maintaining flexibility. Its regressive taper (0.08 at the tip, decreasing to 0.04 coronally) minimizes unnecessary dentin loss and enhances debris elimination. HyFlex EDM One files are manufactured using electrical discharge machining (EDM) applied to controlled memory (CM) wire, allowing the instrument to exist in a martensitic phase at intracanal temperatures [21]. This results in exceptional elastic deformation and enables the instrument to retain canal curvature with minimal deviation [12, 42].

For this investigation, MB canals of extracted human mandibular molars were selected, as they frequently exhibit curvature in both buccolingual and mesiodistal planes, making them a suitable model for evaluating the mechanical effects induced by different instrumentation systems [3, 20]. Using natural teeth rather than acrylic blocks ensures more clinically relevant outcomes by accounting for anatomical variability and dentinal microhardness [43]. Standardization was achieved through apical preparation to size #25, which is commonly recommended for the preparation of narrow and curved root canals [44], and with a working length of 19 mm and occlusal reduction to establish flat reference points [45]. Each file was used for a single canal to prevent instrument fatigue and separation.

Several methodologies have been applied to evaluate canal shaping outcomes, including radiography [46], serial sectioning [47], SEM [48], and micro-computed tomography (micro-CT) [49]. While micro-CT is the most accurate method for in vitro imaging, it was unavailable in this study. CBCT, chosen for its availability and accuracy, provides reliable three-dimensional imaging and quantitative assessment of pre- and post-instrumentation canal morphology. This imaging modality offers a balance between accessibility, non-destructiveness, and accuracy for canal transportation and centering assessment [50].

While statistical analysis revealed a significant difference in overall mesiodistal centering ability between HyFlex EDM One and RCS Rainbow One files, indicating that HyFlex EDM One performed slightly better, with minimal canal transportation, enhanced centering ability, and the least canal straightening. These findings are consistent with its design features and CM-wire manufacturing process. Zarc BlueShaper showed similar shaping behavior. RCS Rainbow One, although effective, demonstrated lower mesiodistal centering ability and no statistically significant difference concerning transportation and canal straightening.

Schneider’s method was employed to measure canal curvature [34], as it is considered among the most accurate, simple, and reliable techniques available. Literature has shown that with increasing curvature, the complexity of canal preparation also increases, and greater deviation is observed in teeth with severe curvature compared to those with moderate curvature [40]. In this study, a wider range of canal curvatures (20–50°) was included compared with previous studies, to better evaluate the performance of newly introduced files in the preparation of severely curved root canals.

The reduction in the Schneider angle after instrumentation, referred to as canal straightening, reflects the inability of an instrument to preserve the original canal anatomy [40]. The decrease in Schneider angle following enlargement can be attributed to the natural tendency of the canal to become less curved, which is consistent with previous findings [39, 40]. Canal straightening was lowest in the HyFlex EDM One group, followed by RCS Rainbow One and Zarc BlueShaper, corresponding to their respective flexibility profiles, although these differences were not statistically significant (*P* >0.05).

Despite these differences, all three systems effectively preserved the original canal morphology and maintained canal transportation within the clinically acceptable limit of 0.3 mm proposed by Wu et al. [51], beyond which apical sealing may be adversely affected. This outcome supports the notion that advancements in alloy processing and instrument design contribute meaningfully to safe and efficient canal shaping [12].

Previous studies have shown that files with a constant taper in their apical third demonstrate better centering than those with progressive tapers [52]. However, this study revealed superior centering with the variable taper designs of HyFlex EDM One and Zarc BlueShaper, potentially due to their metallurgy and flexible design features that allow better navigation of canal curvatures. This discrepancy highlights the influence of thermomechanical properties over taper geometry alone.

The glide path created by Z1, the preliminary instrument in the Zarc system, may have contributed to improved shaping by reducing abrupt diameter transitions between subsequent files. This smoother progression potentially decreases the risk of transportation and improves shaping fluidity, especially in the apical third. The combined effect of the Zarc blue alloy, pre-enlargement shaping using Z1 glide path, and variable taper likely reduced deformation at curvature sites, thereby minimizing straightening as proposed by Greco et al. [15].

The findings of this study align with those of Silva et al., who reported that the RCS Rainbow One file exhibits lower flexibility despite its DLC coating compared to other heat-treated NiTi files [17]. HyFlex EDM One demonstrated superior flexibility and shaping behavior, effectively following the original canal curvature and resulting in better centering ability, which is consistent with the findings reported by Aktaş et al. [53] that may be attributed to its variable taper and heat treatment, which enhance the flexibility and fatigue resistance.

The main strength of this study lies in the evaluation of canal transportation and centering ability in both mesiodistal and buccolingual directions, providing a more comprehensive assessment of root canal preparation in multiple planes. In addition, the inclusion of changes in the angle of curvature allowed for a more precise comparison of the shaping performance among the three file systems. The limitations of this study include the use of CBCT for morphometric evaluation. Although CBCT offers adequate spatial resolution, micro-CT would provide higher-resolution imaging and allow for more precise detection of minute canal modifications and more accurate quantitative results. Furthermore, the study did not assess the extent of uninstrumented canal walls, which is a critical parameter for evaluating debridement efficacy and shaping completeness. A wide range of canal curvatures (20°–50°) was included in this study, and the lack of balanced group distribution may have influenced the shaping outcomes. Also, Inter-individual variability factors, including donor age, dentin hardness, and the degree of dentinal sclerosis, were not standardized in this study and may have affected the shaping outcomes. Additionally, the investigation was limited to mesiobuccal canals of mandibular first molars; therefore, evaluating other anatomically complex canal configurations, such as maxillary second mesiobuccal (MB2) or C-shaped canals, would enhance the generalizability and clinical relevance of the findings.

## Conclusion

Within the limitations of this in vitro study, the null hypothesis was accepted, as no statistically significant differences were detected among the tested systems for canal transportation, buccolingual centering ability, and canal straightening, despite RCS Rainbow One showing a statistically significant lower mesiodistal centering ability. Overall, all three rotary systems were effective in preserving the original canal anatomy and demonstrated acceptable shaping performance in curved root canals.

## Supplementary Information

Supplementary Material 1.

Supplementary Material 2.

Supplementary Material 3.

## Data Availability

The datasets generated and analyzed during the current study are available from the corresponding author upon reasonable request.

## Abbreviations

CBCT: Cone Beam Computed Tomography
CM: Controlled memory
CT: Computed Tomography
DLC: Diamond-Like Carbon
EDM: Electrical Discharge Machining
EDTA: Ethylenediaminetetraacetic acid
KV: Kilovolt
MA: Milliampere
MB: Mesiobuccal
NiTi: Nickel-Titanium
SD: Standard deviation
St-St: Stainless-steel
