# Supplementary figures and images for: Bidirectional evaluation of canal transportation, centering ability and curvature changes of three NiTi rotary systems using cone beam computed tomography (invitro study)
Source: BMC Oral Health. 2025 Dec 17;25:1936. doi: 10.1186/s12903-025-07414-z (PMC12742195; doi:10.1186/s12903-025-07414-z)

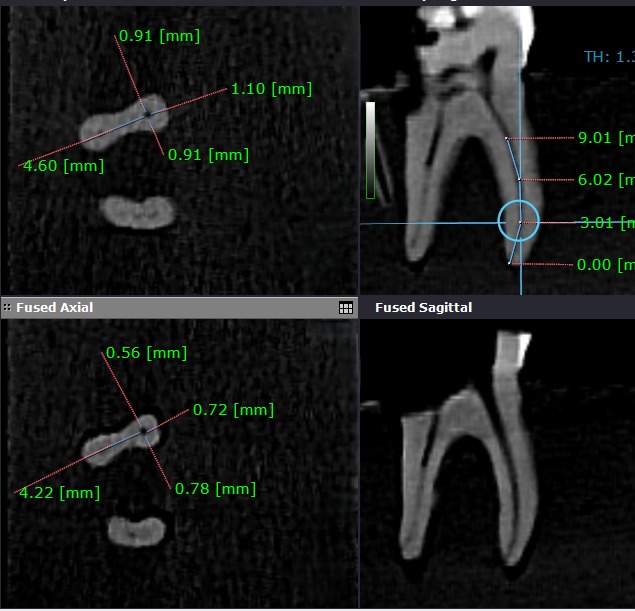

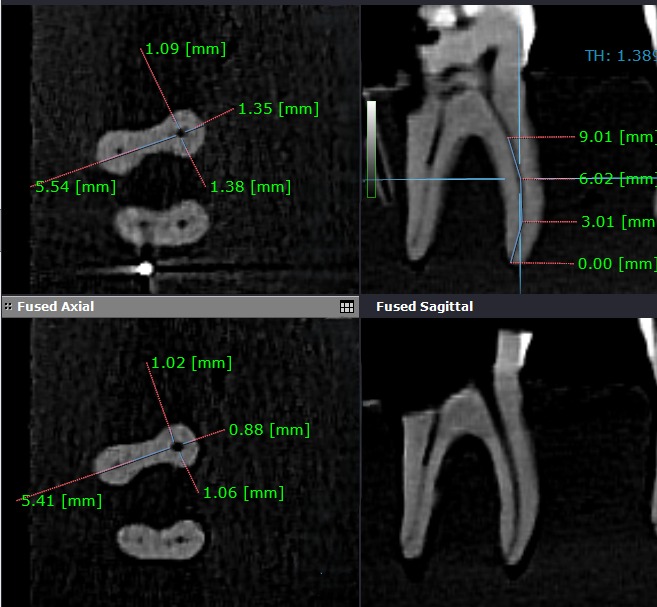
Uncropped images


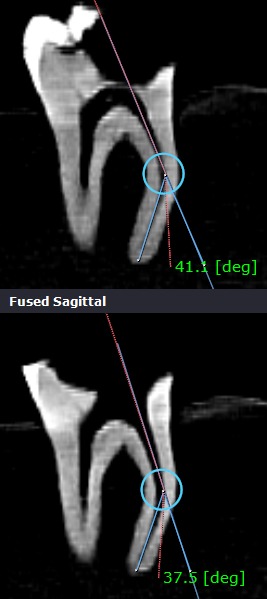

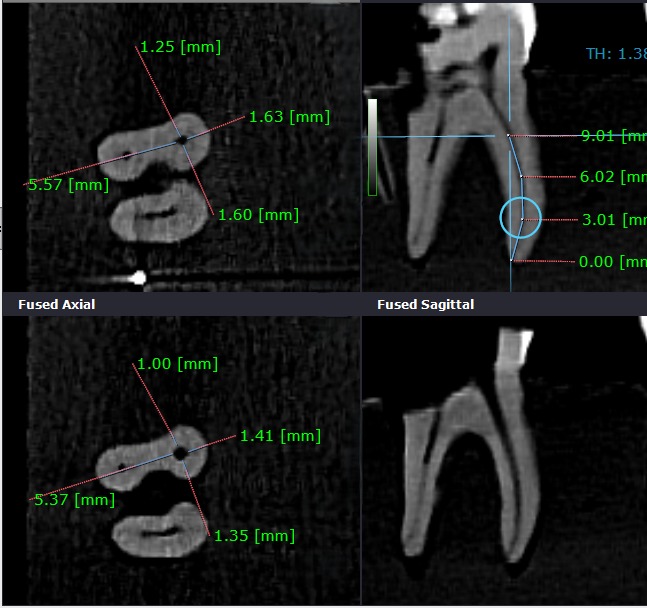

Supplement: Supplementary file 2 — Supplementary Material 2. [file 12903_2025_7414_MOESM2_ESM.docx]
