# Supplementary material for: Bidirectional evaluation of canal transportation, centering ability and curvature changes of three NiTi rotary systems using cone beam computed tomography (invitro study)
Source: BMC Oral Health. 2025 Dec 17;25:1936. doi: 10.1186/s12903-025-07414-z (PMC12742195; doi:10.1186/s12903-025-07414-z)
Supplement: Supplementary file 3 — Supplementary Material 3. [file 12903_2025_7414_MOESM3_ESM.docx]

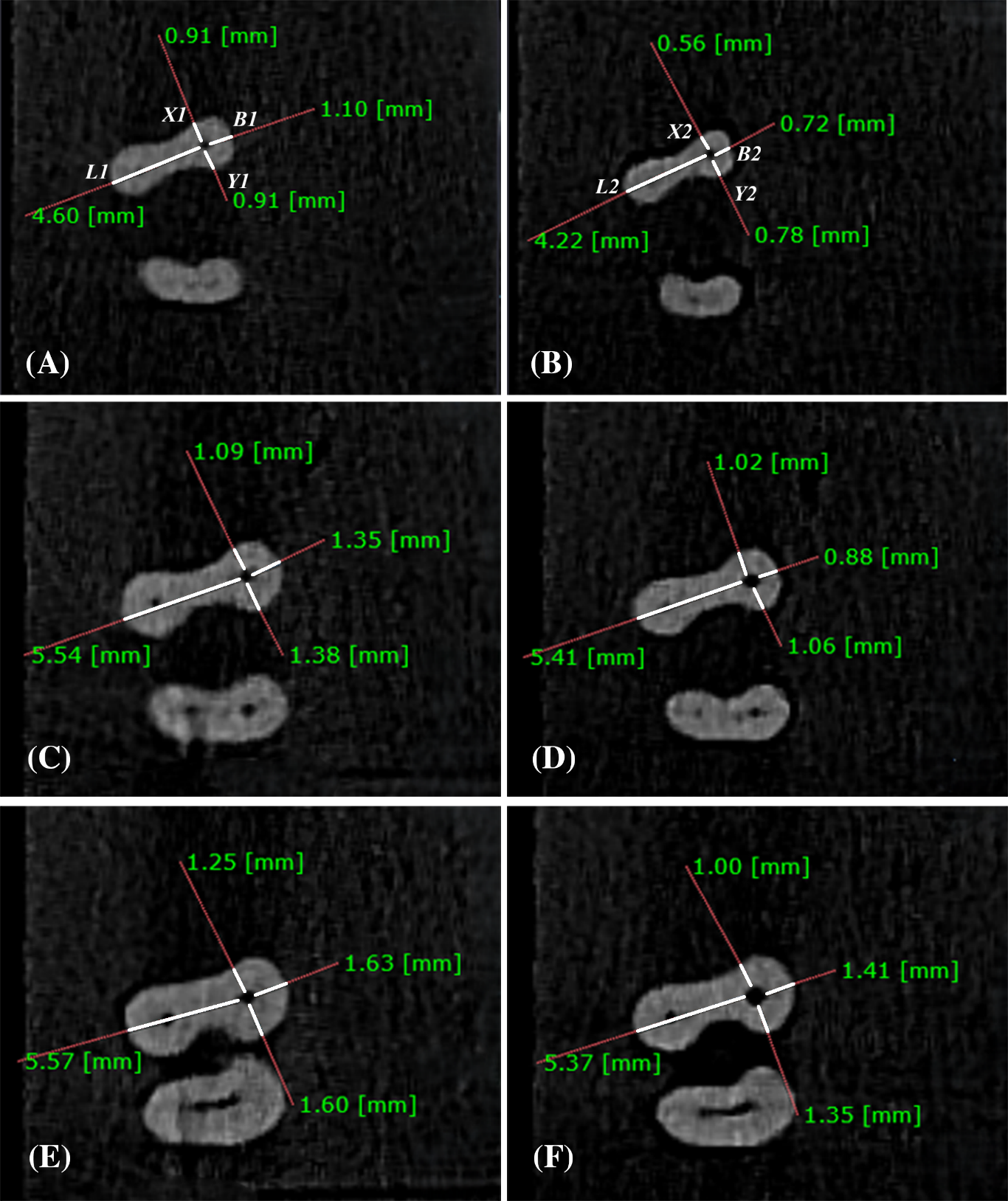


***Figure (1):*** *Showing CBCT images analysis of one of the samples at three, six- and nine-mm levels. (A&B) Axial sections of pre and post instrumentation measurement at three mm level, respectively. (C&D) Axial sections of pre and post instrumentation measurement at six mm, respectively. (E&F) Axial sections of pre and post instrumentation measurement at nine mm, respectively.*

***
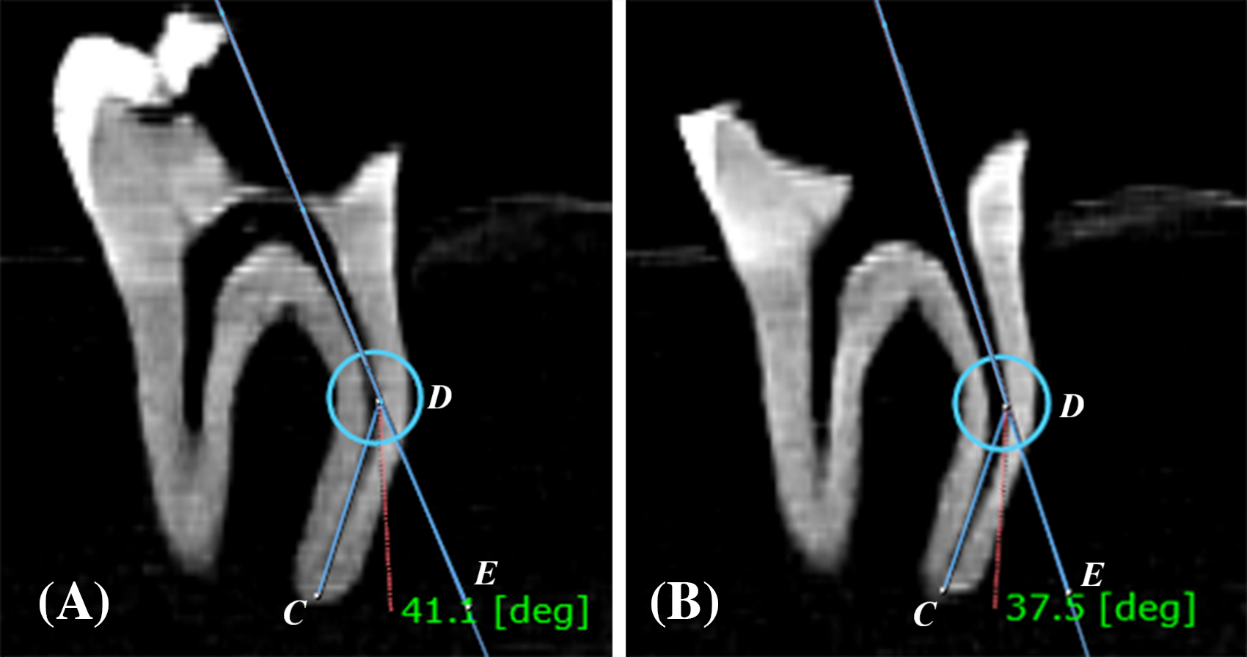
***

***Figure (2):*** *Representing CBCT sagittal view to measure Schneider angle of curvature* ***CDE****. (A) preoperative angle of curvature. (B) postoperative angle of curvature.*
